# Improvement of preharvest sprouting resistance with *MOTHER OF FT AND TFL 1* + mutated *ABA 8ʹ-hydroxylase* in white-seeded durum wheat

**DOI:** 10.1270/jsbbs.22018

**Published:** 2022-12-06

**Authors:** Yusuke Ban, Keita Kato, Miwako Ito, Mikiko Yanaka, Kanenori Takata

**Affiliations:** 1 Western Region Agricultural Research Center, National Agriculture and Food Research Organization (NARO), 6-12-1 Nishifukatsu-cho, Fukuyama, Hiroshima 721-8514, Japan

**Keywords:** *ABA*, *MFT*, preharvest sprouting, *TaABA8ʹOH1*, white-seeded durum wheat

## Abstract

Improvement of preharvest sprouting (PHS) resistance is an important objective in the breeding of durum wheat (*Triticum turgidum* ssp. *durum* (Desf.) Husn.) in Japan, where the harvest timing overlaps with the rainy season. In a previous study, we showed that an *R*-gene associated with red seed color was the most effective at promoting PHS resistance in durum wheat. However, red-seeded durum wheat is not popular because it discolors pasta. Here, to improve PHS resistance without the *R*-gene, we introduced a PHS resistance allele of *MOTHER OF FT AND TFL 1* (*MFT*) and a mutated *ABA 8ʹ-hydroxylase* (*ABA8ʹOH1-A*), which is involved in abscisic acid (ABA) catabolism, singly or together into white-seeded durum wheat. The introduction of both genes reliably and stably improved PHS resistance under all tested conditions. Modification of ABA catabolism might be an effective way to improve PHS resistance in durum wheat. Our findings will contribute to improved PHS resistance in breeding for white-seeded durum wheat.

## Introduction

Durum wheat (*Triticum turgidum* ssp. *durum* (Desf.) Husn.) is an important crop worldwide. Pasta is made from semolina produced by milling of durum wheat. Japan imports almost all of its durum wheat, including about 200 000 t of Canada Western Amber Durum a year from Canada (https://www.maff.go.jp/j/seisan/boueki/mugi_zyukyuu/attach/pdf/index-109.pdf). Domestic production is rare in Japan because the harvest time of durum wheat overlaps with the rainy season, and rainfall at harvest causes preharvest sprouting (PHS), to which durum wheat cultivars are susceptible (Kai *et al.* 1998) and which reduces semolina and pasta quality (Fu *et al.* 2014). Recently, the Western Region Agricultural Research Center (WARC) of the National Agriculture and Food Research Organization developed the first Japanese durum wheat cultivar, ‘Setodure’ (Yanaka *et al.* 2018). ‘Setodure’ is a white-seeded cultivar with low PHS resistance, but its early maturity makes it less likely to encounter rain. ‘Setodure’ production is limited to regions with low precipitation (e.g. Setouchi region), so it is not widely grown in Japan.

In a previous study, we introduced two PHS-resistance genes and a QTL from common wheat (*T*. *aestivum* L.), namely an *R*-gene for red seed color, the *MOTHER OF FT AND TFL 1* (*MFT*) gene, and *Qphs-5AL*, into ‘Setodure’ in different combinations to improve PHS resistance (Kato *et al.* 2017). The *R*-gene, *MFT*, and *QPhs-5AL* all confer PHS resistance in common wheat under a rainy climate in Japan (Himi *et al.* 2011, Kottearachchi *et al.* 2008, Nakamura *et al.* 2011). We found that a red-seeded durum wheat line with all three genes had PHS resistance similar to that of Japanese common wheat cultivars. In contrast, a white-seeded durum wheat line with *MFT* and *Qphs-5AL* (but not the *R*-gene) had poor PHS resistance. Thus, the *R*-gene was the most effective at promoting PHS resistance.

However, red-seeded durum discolors pasta owing to contamination by bran and germ particles (Symons *et al.* 2009). Most wheat used for pasta production is a white-seeded durum wheat called “amber durum”. White-seeded wheat has little discoloration (Kaneko *et al.* 1994). Red-seeded durum wheat has a low value and has long been used in livestock feed (USDA 1933). It has been discontinued in Canada (McCallum and DePauw 2008). As red-seeded durum wheat cultivars are unlikely to be acceptable for pasta production in Japan, we therefore have to find ways to improve PHS resistance without the *R*-gene and its resultant red seed color.

In addition to the genes and QTL used in our previous study (Kato *et al.* 2017), Chono *et al.* (2013) reported that reduced abscisic acid (ABA) catabolism due to mutations in *ABA 8ʹ-hydroxylase* (*TaABA8ʹOH1*), which encodes the enzyme catalyzing the hydroxylation of ABA at the 8ʹ-position, may improve PHS resistance in white-seeded common wheat. They found an insertion mutation on the D genome (*ABA8ʹOH1-D*) and a deletion mutation on the A genome (*ABA8ʹOH1-A*). These findings encouraged us to use *ABA8ʹOH1-A* from common wheat to improve PHS resistance in white-seeded durum wheat, because we have shown that the introduction of genes and QTLs from common wheat by interbreeding improved PHS resistance in durum wheat (Kato *et al.* 2017). A single mutation in *TaABA8ʹOH1-D* had no clear effect on germination inhibition (Chono *et al.* 2013). Thus, we assumed that *ABA8ʹOH1-A* alone might not be sufficient, and chose to add *MFT*, the second most effective gene for improving PHS in durum wheat (Kato *et al.* 2017). We hypothesize that the introduction of *ABA8ʹOH1-A* plus *MFT* can improve PHS resistance in white-seeded durum wheat. Here, we introduced *MFT* and *ABA8ʹOH1-A* singly and together into the white-seeded durum wheat ‘Setodure’ and conducted germination tests with near-isogenic lines (NILs) and ‘Setodure’.

## Materials and Methods

### Plant materials

We developed three NILs with different combinations of *MFT* and *ABA8ʹOH1-A* by backcrossing. The PHS-susceptible ‘Setodure’ was used as the recurrent parent, the bread wheat cultivar ‘Zenkojikomugi’ as the *MFT* donor, and the bread wheat mutant line TM1G1833 as the *ABA8ʹOH1-A* donor. Through the use of DNA markers, we distinguished two alleles in *MFT*, namely resistant allele *Zen* (*MFTz*) and susceptible allele *Seto* (*MFTs*), and two alleles in *ABA8ʹOH1-A*, namely susceptible wild-type allele (*ABA8ʹOH1-Aw*) and resistant mutant-type allele (*ABA8ʹOH1-Am*) (Table 1). We designated the NILs as “+*MFT*” (= MFTz-ABA8ʹOH1-Aw), “+*ABA8ʹOH1-A*” (= MFTs-ABA8ʹOH1-Am), and “+*MFT*+*ABA8ʹOH1-A*” (= MFTz-ABA8ʹOH1-Am). ‘Setodure’ is MFTs-ABA8ʹOH1-Aw. The generation of the NILs used was BC_5_F_4_. We grew ‘Setodure’ and the three NILs in fields at WARC (34°30ʹ4ʺ N, 133°23ʹ12ʺ E) in 2019–20 and 2020–21. The plots were two parallel 16-cm-spaced rows 60 cm in length with a 6-cm planting distance between seeds, with four replications. Plants were sown in November and harvested at full maturity in June. All management followed the standard procedures used at WARC.

### Genotyping of *MFT* and *ABA8ʹOH1-A*

DNA was extracted by the potassium acetate method (Dellaporta *et al.* 1983). *MFT* was genotyped by the methods of Chono *et al.* (2015) and Kato *et al.* (2017), using a cleaved amplified polymorphic sequence (CAPS) marker (Chono *et al.* 2015). DNA templates were amplified with ExTaq DNA polymerase (TaKaRa, Shiga, Japan) and an *MFT* primer set (Chono *et al.* 2015) under conditions described in Kato *et al.* (2017). The PCR fragments were digested by *Cla*I at 37°C for 1 h, separated by agarose gel electrophoresis, and visualized with Gel Red stain (Biotium, Fremont, CA, USA). *ABA8ʹOH1-A* was genotyped by the method of Chono *et al.* (2013) with slight modifications. DNA templates were amplified with the S2A2 primer set (Chono *et al.* 2013) in a total volume of 10 μL containing 100 ng of genomic DNA, 200 μM each dNTP, 0.2 μM each primer, 0.25 U of ExTaqHS DNA polymerase (TaKaRa), and ExTaq buffer (TaKaRa). The amplification conditions used an initial denaturation at 94°C for 1 min; 30 cycles of 94°C for 30 s, 55°C for 30 s, and 72°C for 30 s; and a final extension at 72°C for 5 min. The PCR fragments were separated by polyacrylamide gel electrophoresis and visualized with Gel Red stain (Biotium).

### Germination tests

Germination tests were conducted according to the method of Kato *et al.* (2017) with some modifications. We harvested eight fully mature spikes from each replicate in both years and divided them into two groups. Four spikes were air-dried at 35°C for 1 day; we designated this sample as being in deep dormancy (mature stage). The other four spikes were kept at room temperature (i.e., about 25°C) for a week, and then air-dried at 35°C for 1 day; we designated this sample as being in light dormancy (full-ripe stage). All samples were stored at –20°C until the germination tests. For the tests, all spikes were thawed at room temperature and the seeds were threshed by hand. One hundred seeds from each sample were put on double-layered filter paper (85-mm grade 1; Whatman, Maidstone, UK) in a 90-mm plastic Petri dish, 8 mL of water containing a 0.01% sterilizer (Beflan; Kumiai Chemical Industry Co., Ltd., Tokyo, Japan) was added, and they were incubated at 20°C for 7 days. Germinated seeds were counted every day for 7 days, and we calculated percentage germination (PG) and germination index (GI). As PG, we present the data for the 1st to the 7th day of imbibition (DI). GI was calculated as (Reddy *et al.* 1985):

GI = [(7·*n*_1_) + (6·*n*_2_) + (5·*n*_3_) + (4·*n*_4_) + (3·*n*_5_) + (2·*n*_6_) + (1·*n*_7_)]/(7 × total seeds) × 100

where *n_i_* is the number of germinated seeds on day *i*.

### Measurement of agronomic traits

Heading date and maturity date were determined based on UPOV guideline (https://www.upov.int/edocs/tgdocs/en/tg003.pdf) in the field in 2020 and 2021. For measurement of grain and spike traits, grains were harvested from the plots in 2020 and 2021, and spikes were harvested from the plots in 2021. Grain protein content was analyzed by near infrared spectrophotometer (IM9500, Perten Instruments, Stockholm, Sweden). Grain diameter, thousand kernel weight, and grain hardness were measured with single kernel characterization system (SKCS4100, Perten Instruments). Awn and spike length were measured using a caliper, and number of spikelet and grains per spike were counted manually. Spikelet density was calculated based on spikelet number and spike length.

### Statistical analyses

Before statistical analysis, we evaluated the normality of the distribution of the residual variance of each trait by the Kolmogorov–Smirnov one-sample test (Campbell 1974). Since normality was not rejected at *P* = 0.05, except for the values on 1 DI, we did not transform the data. We used R v. 3.3.3 software (R Core Team 2017) for all statistical analyses. For PG and GI, multiple comparisons among ‘Setodure’ and NILs were done by two-way ANOVA of the effects of genotype and year, followed by Tukey’s honestly significant difference (HSD) test. Variance components for genotype, year, and their interaction were calculated from the mean squares. To assess the contribution of the source of PHS resistance, we also analyzed the datasets by two-way ANOVA of the effects of *MFT* and *ABA8ʹOH1-A*. Variance components for *MFT*, *ABA8ʹOH1-A*, and their interaction were calculated from the mean squares. For data of agronomic traits, multiple comparisons among ‘Setodure’ and NILs were done by one-way ANOVA followed by Tukey’s honestly significant difference (HSD) test.

## Results

### Difference in percentage germination among ‘Setodure’ and NILs

**Fig. 1.**
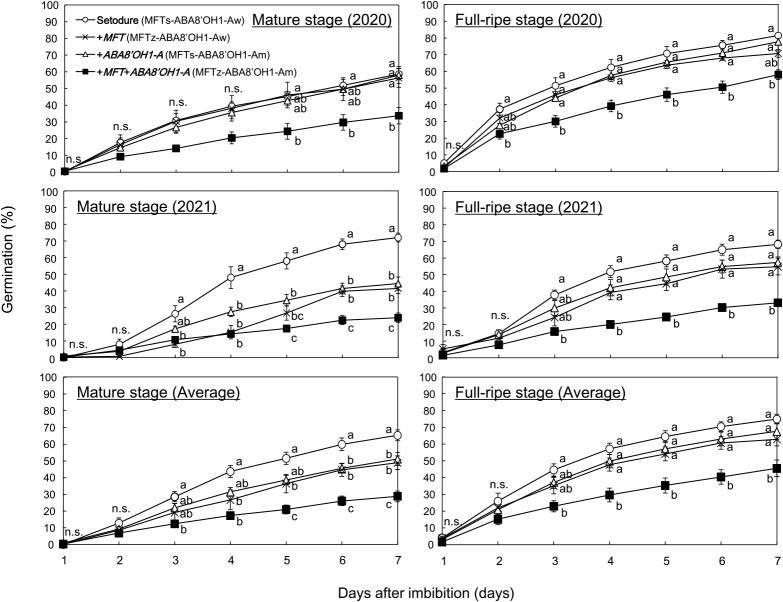
Germination patterns of ‘Setodure’ and NILs at mature (left) and full-ripe (right) stages in 2020 (upper), in 2021 (middle), and averaged over 2 years (lower). Seeds were imbibed at 20°C. Values are means ± SE (*n* = 4 in 2020 and 2021, and *n* = 8 in 2-year average). Plots labeled with the same letter do not differ significantly at *P* < 0.05.

To investigate the effect of the introduction of *MFT* and *ABA8ʹOH1-A*, we examined the germination patterns of ‘Setodure’ and NILs from 1 to 7 DI at 20°C. Differences among genotypes were significant mainly from mid-period (Fig. 1). Only +*MFT*+*ABA8ʹOH1-A* always had lower PG than ‘Setodure’. In 2021 and the 2-year average, the introduction of either *MFT* (+*MFT*) or *ABA8ʹOH1-A* (+*ABA8ʹOH1-A*) gave lower PG than ‘Setodure’ at the mature stage, but values were still higher than those of +*MFT*+*ABA8ʹOH1-A*. There was no significant difference among +*MFT*, +*ABA8ʹOH1-A*, and ‘Setodure’ at the full-ripe stage.

### Difference in germination index among ‘Setodure’ and NILs

**Fig. 2.**
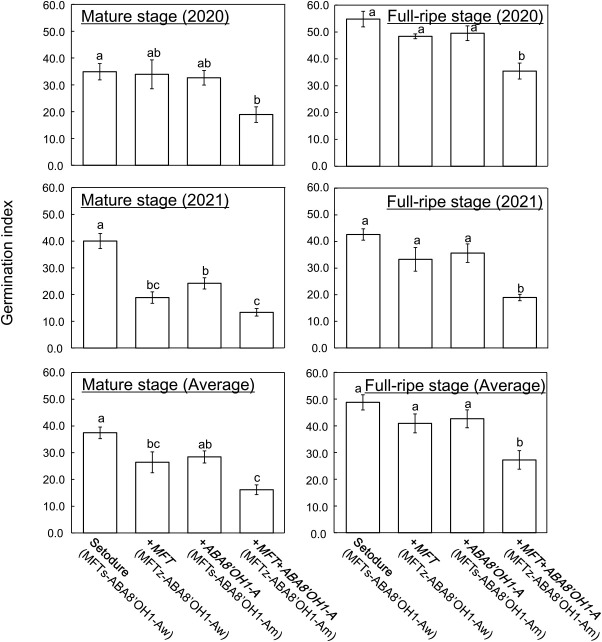
Germination index of ‘Setodure’ and NILs at mature (left) and full-ripe (right) stages in 2020 (upper), in 2021 (middle), and averaged over 2 years (lower). Seeds were imbibed at 20°C. Values are means ± SE (*n* = 4 in 2020 and 2021, and *n* = 8 in 2-year average). Bars labeled with the same letter do not differ significantly at *P* < 0.05.

To confirm the effect of the introduction of *MFT* and *ABA8ʹOH1-A*, we calculated the GI of ‘Setodure’ and NILs at 20°C. As in PG, only +*MFT*+*ABA8ʹOH1-A* always had lower GI than ‘Setodure’ (Fig. 2). In 2021 and the 2-year average, +*MFT* had lower GI than ‘Setodure’ at the mature stage, but not at the full-ripe stage. +*ABA8ʹOH1-A* had lower GI than ‘Setodure’ only in 2021 at the mature stage. These results confirm the effect of the introduction of both *MFT* and *ABA8ʹOH1-A*. There were some differences between 2020 and 2021. In both PG and GI, there was a significant difference between ‘Setodure’ and both +*MFT* and +*ABA8ʹOH1-A* at the mature stage in 2021, but not in 2020 (Figs. 1, 2). It is well known that weather conditions during the grain filling period affect seed dormancy in wheat (Lunn *et al.* 2002, Reddy *et al.* 1985). So the differences between 2020 and 2021 might be due to differences in precipitation and temperature, for example, during the grain filling period.

### Estimation of variance components for *MFT* and *ABA8ʹOH1-A*

To evaluate the contributions of *MFT*, *ABA8ʹOH1-A*, and their interaction on PHS resistance, we conducted two-way ANOVA of their effects using the PG dataset. First, we estimated variance components for genotype, year, and their interaction to find the condition that maximized the variance components of genotype. Values ranged from 17.8% to 56.9% (data not shown), and the percentage was greatest at the mature stage at 7 DI. Next, we analyzed the dataset of the mature stage at 7 DI by two-way ANOVA (Tables 2, 3). The effects of both *MFT* and *ABA8ʹOH1-A* were significant, and the percentage of variance components was 42.5% for *MFT* and 32.9% for *ABA8ʹOH1-A*. *MFT* × *ABA8ʹOH1-A* was not significant. We also estimated the variance components for *MFT* and *ABA8ʹOH1-A* using GI at the mature stage by two-way ANOVA to assess the effect of resistance source. Results were similar (data not shown). The effects of both *MFT* and *ABA8ʹOH1-A* were significant, and the percentage of variance components was 39.7% for *MFT* and 27.1% for *ABA8ʹOH1-A*. *MFT* × *ABA8ʹOH1-A* was not significant. These results indicate that *MFT* and *ABA8ʹOH1-A* had additive effects and contributed independently to PHS resistance.

### Effect of *MFT* and *ABA8ʹOH1-A* introduction on agronomic traits

To assess the effect of *MFT* and *ABA8ʹOH1-A* introduction on agronomic traits, we measured date to heading, date to maturity, grain traits, and spike traits (Tables 4, 5). There was no significant difference between ‘Setodure’ and NILs in date to heading, date to maturity, and grain traits in both tested years (Table 4). For spike traits, spike length of +*MFT*+*ABA8ʹOH1-A* was shorter than that of ‘Setodure’, and spikelet density of +*ABA8ʹOH1-A* and +*MFT*+*ABA8ʹOH1-A* were higher than that of ‘Setodure’ (Table 5). Other spike traits didn’t show significant difference between ‘Setodure’ and NILs (Table 5). In addition, no visible difference between ‘Setodure’ and NILs was observed in the field (i.e., plant height, plant posture, lodging, leaf color, spike color, and disease symptoms; data not shown).

## Discussion

We successfully improved PHS resistance by the introduction of both *MFT* and *ABA8ʹOH1-A* into the white-seeded ‘Setodure’. The effect is reliable, because +*MFT*+*ABA8ʹOH1-A* always had the lowest PHS level (Figs. 1, 2). The introduction of *MFT* alone may be effective (Kato *et al.* 2017), and *ABA8ʹOH1-A* could enhance the effect as in common wheat (Chono *et al.* 2013). However, as we expected, the effects are not stable in durum wheat, because significant effects were detected only at the mature stage in 2021 and the 2-year average (Figs. 1, 2). Similar results for *MFT* were found in our previous study (Kato *et al.* 2017), in which the introduction of *MFT* alone had an effect only on PG at 20°C at the mature stage and on GI at 20°C at the full-ripe stage. Even though *MFT* has a strong effect on grain dormancy in common wheat (Nakamura *et al.* 2011), the introduction of *MFT* alone does not have sufficient effect on PHS, so it should be introduced with other genes or QTLs to improve PHS resistance in durum wheat.

In our previous study, we showed that a NIL combining *MFT* and *QPhs-5AL* had the lowest GI and PG among NILs with white seeds (Kato *et al.* 2017). Here, we didn’t analyze NILs with *QPhs-5AL* because its effect was weak (Kato *et al.* 2017). However, we can compare the contributions of *ABA8ʹOH1-A* and *QPhs-5AL* to PHS from the percentage of variance components of each. The percentage of variance components was 31.6% for *MFT* and 3.0% for *QPhs-5AL*, and the ratio of *QPhs-5AL* to *MFT* was 9.6% (Kato *et al.* 2017). Here, the percentage of variance components was 42.5% for *MFT* and *ABA8ʹOH1-A* for 32.9% (Table 3), and the ratio of *ABA8ʹOH1-A* to *MFT* was 77.3%. The effect of *MFT* was almost the same here as in Kato *et al.* (2017). In contrast, the effect of *ABA8ʹOH1-A* was much higher than that of *QPhs-5AL*, and the ratio of variance components of *ABA8ʹOH1-A* to *MFT* was higher than that of *QPhs-5AL* to *MFT*. There were no epistatic effects between *MFT* and *QPhs-5AL* (Kato *et al.* 2017) or *ABA8ʹOH1-A* (Tables 2, 3). Therefore, we can conclude that the contribution of *ABA8ʹOH1-A* to PHS is much greater than that of *QPhs-5AL*. The combination of *MFT* and *ABA8ʹOH1-A* is the best gene set to improve white-seeded durum wheat at present.

In our program for interbreeding of durum wheat, the introduction of several genes or QTLs from common wheat tends to have unfavorable effects such as reduction of grain yield or quality (data not shown). In this study, we evaluated the effect of *MFT* and *ABA8ʹOH1-A* introduction on agronomic traits. *ABA8ʹOH1-A* might affect on spike length and spikelet density in durum wheat because introduction of *ABA8ʹOH1-A* resulted in shorter spike length and higher spikelet density (Table 5). In contrast, yield components (i.e., grain number per spike and thousand kernel weight) was not significantly different by introduction of *MFT* and *ABA8ʹOH1-A* (Tables 4, 5). Moreover, grain hardness and grain protein content, that are important determinants of semolina yield and pasta quality (Dexter and Matsuo 1977, Hruškova and Švec 2009, Matsuo *et al.* 1972), didn’t show significant difference by introducing *MFT* and *ABA8ʹOH1-A* (Table 5). From the current results, we infer that introduction of *MFT* and *ABA8ʹOH1-A* doesn’t have a negative effect on grain yield and quality. However, introduction of *MFT* and *ABA8ʹOH1-A* tended to reduce thousand kernel weight and grain number per spike (Tables 4, 5). The effect on yield of the introduction both *MFT* and *ABA8ʹOH1-A* should be noted. In 2021, the NILs showed about 5 to 10% reduction of thousand kernel weight compared to ‘Setodure’ although there was no significant difference (Table 4). In 2021, large standard errors (SE) were observed in thousand kernel weight and these affect on statistical analysis. The large SE might be ascribed to small plot size (i.e., 60 cm in length). Thus, continuous yield test with large plot size over several years will be needed to clarify the effect of *MFT* and *ABA8ʹOH1-A* introduction on grain yield.

To evaluate PHS in our wheat breeding program, cultivars and lines are rated on a scale of “easy” to “very difficult” to germinate. In breeding common wheat, lines rated “medium” to “very difficult” are preferable in Japan. In 2020, we compared PG of +*MFT*+*ABA8ʹOH1-A* at 15°C with that of common wheat cultivars. PG of cultivars rated “medium” at 7 DI ranged from 25.0% to 35.0% at the mature stage and from 60.0% to 70.0% at the full-ripe stage. In contrast, PG of +*MFT*+*ABA8ʹOH1-A* at 7 DI was 78.6% at the mature stage and 82.3% at the full-ripe stage. These results indicate that the effect of the introduction of both *MFT* and *ABA8ʹOH1-A* is not enough for production of white-seeded durum wheat in Japan without the threat of PHS. Therefore, further investigation will be needed to find additional effective genes or QTLs for improving PHS in white-seeded durum wheat.

ABA is strongly involved in regulating seed germination and dormancy; high ABA content in seed is associated with low germinability, and thus improves PHS resistance (Cutler *et al.* 2010, Fang and Chu 2008). Three approaches can be used to improve PHS resistance through ABA function: enhancing ABA biosynthesis, reducing ABA catabolism, and enhancing sensitivity to ABA. We assessed the effectiveness of reducing ABA catabolism via modification of *ABA8ʹOH1-A* in durum wheat here. Chono *et al.* (2013) reported that an *ABA8ʹOH1-A* mutant-type line contained more ABA than the wild-type after the middle stages of seed development. Although we did not assess ABA content, our results imply that modification of ABA catabolism could improve PHS resistance in durum wheat as well as in common wheat. In common wheat, a *TaABA8ʹOH1-A*–*TaABA8ʹOH1-D* double mutant had higher PHS resistance than a *TaABA8ʹOH1-D* single mutant (Chono *et al.* 2013). Exploring *ABA8ʹOH1-B* mutant and using both *TaABA8ʹOH1-A* and *TaABA8ʹOH1-B* mutant would be effective to improve PHS resistance in durum wheat. Enhancing ABA biosynthesis would seem to be a simple way to increase ABA content. However, Lang *et al.* (2021) recently reported that *Myb10-D* (*TaMyb10* on 3 DL), which regulates red seed color, enhances ABA biosynthesis via transcription of *9-cis-epoxycarotenoid dioxygenase* (*NCED*), which encodes one of the key enzymes for ABA biosynthesis, and thus confers PHS resistance. Himi *et al.* (2011) reported that *TaMYB10* on 3 AL regulates also red seed color and has a positive effect on PHS resistance. It can be hypothesized that *TaMYB10* upregulates *NCED* expression. Enhancement of ABA biosynthesis in white-seeded durum wheat would be difficult because *MYB10* controls both red seed color and ABA biosynthesis. Sensitivity to ABA could be enhanced by using *TaABI5*, a homolog of Arabidopsis *ABA insensitive 5*, as Utsugi *et al.* (2020) reported that *TaABI5* plays an important role in seed dormancy and PHS in common wheat, and suggested that the expression of *TaABI5* increases sensitivity to ABA. A high-expression allele of *TaABI5* might improve PHS resistance in durum wheat. These findings could lead to further improvement of PHS resistance through ABA function in durum wheat.

Collectively, our results show that (1) the introduction of both *MFT* and *ABA8ʹOH1-A* can improve PHS resistance in white-seeded durum wheat, and (2) the modification of ABA catabolism might be an effective way to improve PHS resistance in durum wheat, as in common wheat. We anticipate that our results will create opportunities for stable production of durum wheat in Japan through improving PHS resistance without red seed color.

## Author Contribution Statement

KT designed the study. KT and MY developed the NILs. YB and KT wrote the initial draft of the manuscript. YB, KK, and MI conducted field trials and germination tests. YB, KK, MI, MY, and KT contributed data analysis and interpretation of data. All authors reviewed and approved the manuscript.

## Figures and Tables

**Table 1.** Sources of PHS resistance introduced into white-seeded durum wheat ‘Setodure’

Source of resistance (chromosome position)	Selection marker (marker type)	Alleles		Reference
		Resistant	Susceptible	
*MFT* (3AS)	CS3A06Proseq-F3,R5 (CAPS)	Zen (MFTz)	Seto (MFTs)	Nakamura *et al.* (2011)
*TaABA8ʹOH1-A* (6A)	S2A2 (deletion)	Mutant-type (ABA8ʹOH1-Am)	Wild-type (ABA8ʹOH1-Aw)	Chono *et al.* (2013)

**Table 2.** ANOVA of percentage germination at mature stage over 7 days after imbibition in 2 years

Source of variation	Degrees of freedom	Mean squares	*P*-value	Expected mean squares
*MFT*	1	3008.4	1.16E-05	σ^2^ + 8σ_(MFT×ABA8__ʹ__OH1-A)_^2^ + 16σ_MFT_^2^
*ABA8ʹOH1-A*	1	2342.1	6.40E-05	σ^2^ + 8σ_(MFT×ABA8__ʹ__OH1-A)_^2^ + 16σ_ABA8__ʹ__OH1-A_^2^
*MFT* × *ABA8ʹOH1-A*	1	70.7	0.422	σ^2^ + 8σ_(MFT×ABA8__ʹ__OH1-A)_^2^
Residuals	28	106.3		σ^2^

**Table 3.** Estimates of variance components and their proportion of the total obtained from ANOVA of percentage germination at mature stage over 7 days after imbibition in 2 years

Variance component	Estimate	Percentage estimate (%)
σ*_MFT_*^2^	183.61	42.5
σ*_ABA8__ʹ__OH1-A_*^2^	141.96	32.9
σ_(_*_MFT_*_×_*_ABA8__ʹ__OH1-A_*_)_^2^	0.00*^a^*	0.0
σ_Residuals_^2^	106.30	24.6
σ_Total_^2^	431.87	100.0

*^a^* Negative values were assumed to be 0.

**Table 4.** Date to heading, date to maturity, and grain traits of ‘Setodure’ and NILs in 2020 and 2021

Genotype	Date to heading (days)	Date to maturity (days)	Grain traits			
			Protein content (%)	Diameter (mm)	Thousand kernel weight (g)	Hardness (HI)
2020						
Setodure (MFTs-ABA8ʹOH1-Aw)	157.3 ± 0.25 a	210.0 ± 0.00 a	12.4 ± 0.2 a	3.06 ± 0.01 a	48.3 ± 0.7 a	95.6 ± 1.1 a
+*MFT* (MFTz-ABA8ʹOH1-Aw)	157.0 ± 0.00 a	210.0 ± 0.00 a	12.1 ± 0.3 a	3.02 ± 0.02 a	46.1 ± 0.5 a	96.5 ± 2.0 a
+*ABA8ʹOH1-A* (MFTs-ABA8ʹOH1-Am)	157.3 ± 0.25 a	210.0 ± 0.00 a	12.8 ± 0.1 a	2.97 ± 0.03 a	46.7 ± 0.4 a	96.2 ± 1.1 a
+*MFT*+*ABA8ʹOH1-A* (MFTz-ABA8ʹOH1-Am)	157.8 ± 0.25 a	210.0 ± 0.00 a	12.7 ± 0.1 a	2.95 ± 0.04 a	46.4 ± 1.5 a	92.7 ± 0.9 a
2021						
Setodure (MFTs-ABA8ʹOH1-Aw)	156.0 ± 0.00 a	204.0 ± 0.00 a	11.9 ± 0.8 a	3.20 ± 0.10 a	53.3 ± 3.3 a	85.4 ± 3.9 a
+*MFT* (MFTz-ABA8ʹOH1-Aw)	156.0 ± 0.00 a	203.8 ± 0.25 a	12.3 ± 0.6 a	3.18 ± 0.02 a	50.5 ± 1.1 a	88.2 ± 3.3 a
+*ABA8ʹOH1-A* (MFTs-ABA8ʹOH1-Am)	156.0 ± 0.00 a	203.3 ± 0.25 a	11.8 ± 0.7 a	3.07 ± 0.03 a	49.4 ± 1.5 a	86.8 ± 4.4 a
+*MFT*+*ABA8ʹOH1-A* (MFTz-ABA8ʹOH1-Am)	156.0 ± 0.00 a	203.8 ± 0.25 a	11.5 ± 0.8 a	3.02 ± 0.04 a	47.5 ± 1.8 a	91.1 ± 1.8 a

Note: Values are mean ± standard error (*n* = 4). Data in the same column with the same letter do not differ significantly at *P* < 0.05.

**Table 5.** Spike traits of ‘Setodure’ and NILs in 2021

	Awn length (cm)	Spikelet number	Spike length (cm)	Spikelet density^*^	Grain number per spike
Setodure (MFTs-ABA8ʹOH1-Aw)	4.4 ± 0.6 a	26.0 ± 0.5 a	9.6 ± 0.3 a	2.73 ± 0.04 c	68.0 ± 3.6 ab
+*MFT* (MFTz-ABA8ʹOH1-Aw)	4.7 ± 0.4 a	27.1 ± 0.4 a	9.4 ± 0.1 a	2.88 ± 0.04 bc	77.6 ± 3.0 a
+*ABA8ʹOH1-A* (MFTs-ABA8ʹOH1-Am)	4.8 ± 0.2 a	26.6 ± 0.3 a	9.0 ± 0.1 ab	2.95 ± 0.03 ab	66.0 ± 2.5 ab
+*MFT*+*ABA8ʹOH1-A* (MFTz-ABA8ʹOH1-Am)	5.1 ± 0.2 a	26.4 ± 0.4 a	8.5 ± 0.2 b	3.10 ± 0.06 a	61.1 ± 3.4 b

Note: Values are mean ± standard error (*n* = 8 for spike). Data in the same column with the same letter do not differ significantly at *P* < 0.05. * Spikelet density = Spikelet number/Spike length.
